# Validation and Development of Claims-Based Algorithms for Identifying Thyroid Eye Disease Using the IRIS Registry-Komodo Linked Database

**DOI:** 10.3390/jcm15103836

**Published:** 2026-05-15

**Authors:** Junjie Ma, Wendy W. Lee, Maurice Alan Brookhart, Madhura A. Tamhankar, Juan Ayala-Haedo, Fang He, Haridarshan Patel

**Affiliations:** 1 Center for Observational Research, Amgen, Inc., Thousand Oaks, CA 91320, USA; majunjie360@gmail.com (J.M.);; 2 Bascom Palmer Eye Institute, Miller School of Medicine, University of Miami, Miami, FL 33136, USA; 3 Department of Population Health Sciences, Duke University School of Medicine, Durham, NC 27701, USA; 4 Department of Ophthalmology, Scheie Eye Institute, Perelman School of Medicine, University of Pennsylvania, Philadelphia, PA 19104, USA; 5 Medical Affairs, Amgen, Inc., Thousand Oaks, CA 91320, USA

**Keywords:** thyroid eye disease, algorithm validation, claims data

## Abstract

**Objectives**: To validate claims-based algorithms for identifying thyroid eye disease (TED) cases and assess whether machine learning can improve case identification in a large, linked real-world dataset. **Methods**: Using a large, linked database from Komodo Health^®^ and Academy IRIS^®^ Registry, we evaluated six rule-based algorithms incorporating Graves’ disease (GD), eye symptoms and signs. The IRIS Registry’s curated data, based on confirmed TED diagnoses from medical notes, served as the reference standard. Additionally, we developed supervised machine learning models using demographic, diagnostic, procedural, and medication data. Feature selection was performed using recursive feature elimination to rank predictive codes and construct a simplified, interpretable model. Cross-validation was used to assess model performance and compare performance with the rule-based algorithms. **Results**: The rule-based algorithms demonstrated a trade-off between sensitivity and specificity, with some achieving high specificity but limited sensitivity. Algorithm 1 had the highest sensitivity (48.7%) but lower specificity (59.9%) and PPV (75.8%). Algorithms 2–5 demonstrated higher specificity (87.2–93.5%) but lower sensitivity (17.8–27.0%). Algorithm 6 improved sensitivity (33.4%) compared to Algorithms 2–5 while maintaining high specificity (86.8%) and a strong PPV (86.7%). Machine learning models demonstrated similar trade-offs. One model achieved improved specificity (77.2%) with sensitivity of 49.3%, outperforming Algorithm 1 in specificity while matching its sensitivity. Another model maximized specificity (91.7%) and PPV (89.8%) at a reduced sensitivity of 28.5%. These results highlight the flexibility of machine learning models in adjusting performance to address different research objectives. **Conclusions**: This study evaluated existing rule-based algorithms for identifying TED cases in claims data, revealing trade-offs between sensitivity and specificity. Machine learning models provide additional flexibility, allowing performance to be tailored to specific research use cases. While no single method consistently outperformed others across all metrics, both rule-based and machine learning approaches demonstrated value in improving TED case identification using real-world data sources.

## 1. Introduction

Thyroid Eye Disease (TED), also known as Graves’ orbitopathy, is a debilitating autoimmune condition that affects approximately 25% to 40% of patients with Graves’ disease [1]. Characterized by inflammation and tissue expansion within the orbit, TED can lead to symptoms ranging from proptosis and diplopia to compressive optic neuropathy and vision loss. The condition has a substantial impact on patients’ quality of life and presents considerable challenges in both diagnosis and management [2]. Despite its clinical importance, TED remains difficult to study in large-scale administrative claims databases due to limitations in diagnostic coding and the lack of specific International Classification of Diseases (ICD) codes for TED [3]. Accurate identification of TED in real-world data has important clinical and public health implications, as it enables more reliable evidence generation to assess disease burden, treatment patterns, outcomes, and healthcare utilization, and to inform clinical and policy decision-making.

Administrative claims data are an important resource for conducting observational research and generating real-world evidence. However, accurate identification of disease cohorts within claims datasets depends on consistent and accurate diagnosis, procedure, and medication codes in claims. As noted, this process is especially challenging for TED due to the lack of specific ICD codes and coding overlap with other thyroid and orbital disorders, which can result in misclassification and potential bias in research findings.

Several observational studies have been conducted in TED using claims-based algorithms [4,5,6,7]. These algorithms often vary in complexity and inclusion criteria, but none have undergone formal validation using a clinically confirmed reference standard. As a result, the operating characteristics of these algorithms remain uncertain, raising concerns about the validity and generalizability of findings based on them. This lack of formal validation represents a key evidence gap, limiting confidence in the use of claims-based algorithms for identifying TED in real-world studies.

Recent advances in computational methods, including machine learning, have shown promise in developing more accurate case-identification algorithms in administrative claims data [8,9]. Machine learning approaches can leverage high-dimensional data to identify complex, nonlinear patterns that traditional rule-based algorithms may miss. However, these techniques require well-annotated datasets with confirmed disease status for training and evaluation.

To address these challenges, we developed and validated claims-based algorithms for identifying patients with TED using a novel linked dataset combining the American Academy of Ophthalmology (Academy) Intelligent Research in Sight (IRIS^®^) Registry and Komodo Health^®^ data. The Academy IRIS^®^ Registry is the largest ophthalmology-specific electronic health record (EHR) enabled registry in the United States (US), which contains structured clinical data and concepts curated from medical notes, and Komodo Health^®^ data, which offers a comprehensive, longitudinal view of US patient journeys across 330 million individuals from over 150 all-payer claims databases. These note-derived concepts from the IRIS Registry enable precise identification of clinically confirmed TED and non-TED patients, which can serve as a reference standard for evaluating claims-based algorithms.

This linkage provided a unique opportunity to assess the real-world performance of existing claims-based algorithms and to explore the potential of machine learning to enhance TED case identification. While machine learning has shown promise in other domains for improving classification accuracy, its effectiveness in identifying TED from administrative claims data remains unknown.

The aim of this study was to evaluate rule-based algorithms for identifying TED from claims data by assessing their diagnostic performance, and to develop and evaluate machine learning-based models to explore improvements in TED case identification. In addition, this study characterized the demographic and clinical profiles of confirmed TED patients, which offered additional insights into TED.

## 2. Methods

### 2.1. Data Sources

The linked data from IRIS Registry and Komodo Health^®^ were used in this study to identify and validate claims-based algorithms for TED. The IRIS Registry, established in 2014, is the world’s largest comprehensive ophthalmology registry, encompassing data on more than 800 million patient visits and including approximately 15,000 ophthalmologists and clinicians in their practices across the US.

The IRIS Registry data were linked to Komodo Research Dataset, one of the largest curated sources of de-identified medical and pharmacy claims data in the US. Komodo Research Dataset provides de-identified, longitudinal claims data that capture detailed healthcare journeys for more than 330 million individuals in the US, integrating data from a wide range of payer and provider sources. The Komodo data available for this linkage represented an enriched sample of patients with eye-related conditions.

TED status in the IRIS Registry was determined using a clinically validated natural language processing (NLP) model developed by Verana Health [analytics partner of the American Academy of Ophthalmology (AAO) for the IRIS Registry]. The model extracts TED-related clinical concepts from unstructured ophthalmology notes and was validated against manually reviewed clinical documentation. Details of the NLP framework and validation approach have been previously described [10]. The model demonstrated a precision of 0.90, recall (sensitivity) of 0.91, and F1-score of 0.91 for confirmed TED diagnoses, with a reported false positive rate of 23.1% (specificity ≈ 76.9%). The NLP-derived TED classification served as the reference standard for evaluating the performance of claims-based rule-based algorithms and machine learning models in this study.

Patient linkage was conducted using a secure, HIPAA-compliant tokenization process certified by the third-party vendor Datavant. The process enabled accurate matching of patient records across the IRIS Registry and Komodo datasets while maintaining strict patient privacy protections. The final linked dataset comprised 219,942 unique patients.

### 2.2. Study Population

This study included patients who (1) were aged ≥18 years as of the index date, defined as the date of the first TED diagnosis record in the IRIS Registry; (2) had at least 12 months of continuous enrollment in Komodo Research Dataset prior to the index date; and (3) had medical notes evaluated for TED status within the IRIS Registry data. Patients not meeting these criteria were excluded from analysis. These inclusion criteria were selected to ensure adequate baseline claims history for characterization of patient characteristics and to enable use of the IRIS Registry note-derived TED classification as the reference standard.

### 2.3. Identification of TED-Related Codes and Coding Patterns

To characterize TED-related coding patterns, we extracted and analyzed diagnosis, procedure, and medication codes from the Komodo Research Dataset. This included ICD-9/10 diagnosis codes, National Drug Codes (NDC), Healthcare Common Procedure Coding System (HCPCS), and Current Procedural Terminology (CPT) codes. We examined the frequency and distribution of these codes among confirmed TED patients, as well as their temporal proximity to the index date. Specifically, we assessed code occurrences within 90, 180, and 365 days before, on, or after the index date.

### 2.4. Claims-Based TED Identification Algorithms

We evaluated six claims-based algorithms to identify patients with TED using administrative claims data (Table 1). Algorithm development was informed by prior literature and clinical expert input, incorporating combinations of diagnosis codes for hyperthyroidism and TED-related eye symptoms/signs. Algorithm 1 identified patients based solely on the presence of TED-related eye symptoms/signs, without requiring a hyperthyroidism diagnosis. Algorithms 2 through 5 required at least one claim for hyperthyroidism and one for a TED-related eye condition within a 12-month period, with each algorithm varying the specific set of eye symptoms included. Algorithm 6 was similar to Algorithm 2 in symptom criteria but did not impose any temporal constraint between hyperthyroidism and eye condition claims.

### 2.5. Development of a Machine Learning–Based Algorithm

To evaluate whether machine learning methods could further improve claims-based identification of TED, we developed a supervised classification model using the linked IRIS Registry-Komodo dataset. This approach allowed assessment of complex patterns across high-dimensional claims data that may not be fully captured by predefined rule-based algorithms. All diagnosis and procedure codes available in the Komodo Research Dataset were extracted for patients with a clinically validated TED or non-TED status based on structured clinical concepts derived from the IRIS Registry. From the full set of available codes, we identified 4233 diagnosis and procedure codes during the 2-year baseline period that met a minimum prevalence threshold of 0.1% in both TED and non-TED patients. This strategy is consistent with high-dimensional, data-driven variable selection strategies commonly used in epidemiologic research [11].

In addition to the data-driven selection process, a manually curated list of 168 diagnosis codes provided by clinical experts was reviewed. These 168 codes included those associated with TED-related symptoms, signs, and known thyroid comorbidities, including Graves’ disease. After accounting for overlap codes with the data-driven list, the final analytical code list comprised 4358 clinically relevant diagnosis and procedure codes. The combined feature set, composed of both data-driven and expert-curated codes, was used to train a CatBoost classifier. CatBoost is a sparsity-aware, gradient boosting algorithm specifically designed to handle categorical variables, making it suitable for modeling healthcare claims data [9].

To improve the utility and interpretability of the model while reducing the model complexity, we applied a recursive feature elimination (RFE) step to iteratively remove less informative features based on model performance (AUC) [12]. At each iteration, the least informative features were dropped, and model performance was re-estimated using 5-fold stratified cross-validation. The elimination process continued until further feature removal no longer improved cross-validated AUC, and the subset of features yielding the highest average AUC across folds was selected as the optimal set.

In addition, hyperparameter tuning was performed in parallel, resulting in the construction of 81 models. For each model, RFE identified a subset of medical codes (features) that maximized cross-validated AUC. We then tracked which medical codes appeared most consistently across these models. Medical codes that were consistently retained across multiple independent RFE runs and recurred across different model configurations were considered robust predictors and retained for modeling. The final set of consistent medical codes was then used to develop a parsimonious machine learning model for further evaluation and comparison.

To improve interpretability, we excluded certain ICD-10 codes in X category (external causes of morbidity and mortality), Y (external causes of morbidity and mortality), and Z (factors influencing health status and contact with health services). Because these codes reflect healthcare utilization or external events rather than underlying disease, their inclusion could introduce bias and reduce the clinical relevance of the model.

The TED classification status derived from the IRIS Registry served as the reference standard for training and reporting model performance. Features selected through recursive feature elimination were reviewed to confirm clinical plausibility and consistency with expert-defined TED characteristics. This machine learning workflow was designed to build a structured and interpretable claims-based classification model, integrating statistically informative features with clinically validated inputs to support robust identification of TED in observational research.

### 2.6. Performance Evaluation

All rule-based algorithms and machine learning models were evaluated using a standardized set of diagnostic performance metrics to assess their accuracy in identifying patients with TED. Performance evaluation assumed that the IRIS Registry note-derived TED classification served as the reference standard for evaluating the performance of both rule-based and machine learning-based approaches. These metrics included AUC, sensitivity, specificity, positive predictive value (PPV), negative predictive value (NPV), positive likelihood ratio (PLR), negative likelihood ratio (NLR), and F1-score. Together, these measures provided a comprehensive evaluation of each method’s ability to distinguish between TED and non-TED cases, while capturing the trade-offs between false positive and false negative classifications.

As noted, TED status, used as the reference standard for performance evaluation, was derived from the IRIS Registry using a clinically validated NLP algorithm applied to unstructured electronic health record notes. This structured classification served as the reference standard for benchmarking both rule-based and machine learning–based approaches. However, this reference standard is subject to misclassification, including a reported false positive rate of 23.1% (specificity ≈ 76.9%), indicating that some degree of outcome misclassification may be present. This should be considered when interpreting sensitivity and specificity estimates of claims-based algorithms.

For rule-based algorithms, performance metrics were computed based on whether patients met predefined code-based criteria within the claims data. For machine learning models, predicted probabilities of TED were generated, and classification thresholds were applied to assign TED or non-TED labels. Multiple probability thresholds were evaluated to illustrate the trade-off between sensitivity and specificity across different operating points, rather than to identify a single universally optimal cutoff. This approach was intended to reflect that the preferred threshold may vary depending on the research objective and intended application.

In the final cohort that satisfied the study criteria, participants were randomly divided into a 50% training set and a 50% test set. The sampling ratio was selected to ensure that the test set alone would satisfy the minimum sample size requirement (*n* = 1291 TED-positive patients), calculated for estimating sensitivity based on an expected sensitivity of 70%, a 95% confidence level, and a maximum margin of error of 2.5%. Given the observed TED prevalence (~72%) in this enriched linked cohort, the resulting test set size substantially exceeded the minimum total sample required to achieve this number of positive cases. The test set was reserved exclusively for final evaluation of machine learning model performance and was not used during model development.

## 3. Results

### 3.1. Baseline Characteristics

A total of 103,309 adult patients whose medical notes were evaluated for TED were identified in the linked IRIS Registry-Komodo dataset between 1 January 2016, and 30 September 2024. Based on the reference standard derived from medical notes in the IRIS Registry data, 74,223 patients had clinically confirmed TED and 29,086 patients were confirmed non-TED. Following the application of the inclusion criterion requiring at least 365 days of continuous enrollment in Komodo Research Dataset prior to the index date, the final cohort comprised 92,150 patients. The patient selection flow is shown in Figure S1. Of these, 66,396 were confirmed TED cases and 25,754 were confirmed non-TED cases. Table 2 presents the baseline characteristics of confirmed TED and non-TED patients.

The mean Charlson Comorbidity Index (CCI) score, based on claims data, was 2.7 in both the TED and non-TED cohorts. Scores derived from the IRIS Registry data were higher, with a mean of 5.1 in TED patients and 4.1 in non-TED patients, reflecting the greater clinical detail captured in electronic health records.

Similar prevalence of baseline comorbidities was observed between TED and non-TED patients included in this validation study based on claims data. As an ophthalmology EHR-enabled registry, the IRIS Registry provided substantially greater capture of eye-related conditions compared to claims. For example, dry eye was recorded in 11.1% of TED patients and 12.5% of non-TED patients in Komodo Research Dataset, whereas the IRIS Registry captured dry eye in 32.8% and 35.4% of TED and non-TED patients, respectively. Similarly, ocular hypertension was identified in 1.7% of TED patients and 1.5% of non-TED patients in claims data, compared to 4.2% and 3.4% in IRIS Registry data.

### 3.2. TED-Related Codes and Coding Patterns

We examined the frequency and timing of ICD-9/10 diagnosis codes, NDC, HCPCS, and CPT procedure codes among 66,396 confirmed TED patients. Code utilization was assessed within 90, 180, and 365 days before and after the index date to evaluate temporal patterns of diagnostic and treatment activity surrounding the confirmed TED diagnosis.

As shown in Table 3, the most frequently recorded codes included outpatient visits, endocrine- or ophthalmology-related diagnoses, and thyroid function tests. The CPT code 99214 (office/outpatient visit, established patient, moderate complexity) was the most commonly observed procedure following CPT code 99213 (Office/outpatient visit, established patient, low complexity).

Among diagnosis codes, E05.00 (thyrotoxicosis with diffuse goiter without thyrotoxic crisis) appeared in 32.3% of patients within 90 days around the index date and 42.4% within 365 days around the index date. Other frequently observed diagnosis codes included I10 (essential hypertension), E03.9 (hypothyroidism, unspecified), and E78.5 (hyperlipidemia, unspecified).

Ophthalmic codes were also consistently recorded, reflecting clinical evaluation and management of TED. These included H04.123 (dry eye syndrome of bilateral lacrimal glands), 92014 (comprehensive eye exam, established patient), and H05.20 (unspecified exophthalmos), each showing substantial frequency across all time windows surrounding the index date.

Thyroid function testing was also common. The CPT code 84443 (thyroid stimulating hormone test) was observed in 19.0% of patients within 90 days around the index date, 25.2% within 180 days around the index date, and 32.6% within 365 days around the index date. Additional lab codes such as 85025 (complete blood count with differential) and 80053 (comprehensive metabolic panel) showed similar frequency patterns across the intervals.

### 3.3. Performance of Claims-Based TED Algorithms

Six rule-based algorithms for identifying TED cases in administrative claims data were evaluated, using the medical notes–derived TED status from the IRIS Registry as the reference standard. Algorithms requiring hyperthyroidism and TED-related symptoms/signs within a 12-month window (Algorithms 2–5) generally demonstrated high specificity (≥87.2%) and PPVs (ranging from 84.5% to 87.6%), with corresponding PLR values of 1.21 to 2.74 and NLR values of 0.77 to 0.88, but consistently low sensitivity (17.8–27.0%). Algorithm 2 achieved the highest specificity among this group (90.4%), with a sensitivity of 23.6%, PLR of 2.47, NLR of 0.84, and an F1-score of 0.37.

Algorithm 1, which applied a broader set of TED-related codes without requiring hyperthyroidism, showed the highest sensitivity (48.7%) but at the expense of lower specificity (59.9%) and a PPV of 75.8%, with a PLR of 1.21 and an NLR of 0.86. Similar to Algorithms 2–5, Algorithm 6 required the presence of both hyperthyroidism and TED-related eye symptoms/signs but did not impose the restriction of a 12-month time window between the two. It demonstrated a more balanced performance, with a sensitivity of 33.4%, specificity of 86.8%, PPV of 86.7%, PLR of 2.54, NLR of 0.77, and the highest F1-score (0.48) among Algorithms 2–6. The diagnostic performance of each algorithm is presented in Table 4.

### 3.4. Machine Learning Algorithms for TED Identification

From an initial pool of 4358 diagnosis and procedure codes identified based on differential prevalence between patients with TED and patients without TED, recursive feature elimination was used to identify a parsimonious set of predictive features. A total of 81 CatBoost models were trained, each limited to 201 features selected through five-fold cross-validation. From this set, the top 29 codes were selected based on their contribution to cross-validated model performance under tuned hyperparameter settings, with feature importance recalculated at each iteration. Code selection was driven by incremental improvements in model performance (AUC), and the final codes were reviewed to ensure clinical coherence with established TED-related knowledge (Table 5).

The full CatBoost model, which incorporated 4358 diagnosis and procedure codes as input features, demonstrated variable performance across classification thresholds used to classify patients as TED or non-TED (Table 6). At a threshold of 0.60, the model achieved high sensitivity (86.6%) but low specificity (29.9%). As the threshold increased, sensitivity declined while specificity and PPV improved. At a threshold of 0.75, the model yielded a sensitivity of 49.0%, specificity of 78.4%, PPV of 85.4%, NPV of 37.3%, PLR of 2.27, NLR of 0.65, and an F1-score of 0.62. The highest specificity (92.5%) and PPV (90.5%) were observed at the 0.85 threshold, though sensitivity declined to 27.7%. Using the 29 selected features, a simplified CatBoost model with 10 decision trees was developed. At the 0.75 threshold, this model achieved a sensitivity of 49.3%, specificity of 77.2%, PPV of 84.8%, NPV of 37.1%, PLR of 2.16, NLR of 0.66, and an F1-score of 0.62.

## 4. Discussion

This study evaluated and compared the performance of six rule-based algorithms and a supervised machine learning model for identifying TED in administrative claims data. The algorithms were validated against clinically confirmed TED status derived from medical notes in AAO IRIS Registry.

The best performing rule-based algorithm (Algorithm 6), which required the presence of both hyperthyroidism and TED-related eye symptoms or signs, demonstrated the most favorable balance of high specificity (86.8%), high PPV (86.7%), and improved sensitivity (33.4%) relative to other rule-based approaches. Although this algorithm showed improved sensitivity compared to Algorithms 2–5, sensitivity remained modest, indicating that traditional rule-based definitions may miss a substantial proportion of true TED cases.

The CatBoost model, trained on 4358 diagnosis and procedure codes, demonstrated improved performance flexibility through threshold tuning. At a classification threshold of 0.75, the model achieved a sensitivity of 49.0%, specificity of 78.4%, and PPV of 85.4%. At a higher threshold of 0.85, specificity and PPV increased to 92.5% and 90.5%, respectively, while sensitivity declined to 27.7%. These results highlight the model’s adaptability depending on whether greater emphasis is placed on sensitivity or specificity.

To enhance interpretability and facilitate practical application, we developed a simplified version of the CatBoost model using a reduced feature set of 29 codes. These codes were selected through recursive feature elimination from the broader set of 4358 features based on frequency of selection, contribution to model performance, and clinical relevance. The simplified CatBoost model, using only these 29 codes and 10 decision trees, achieved performance metrics comparable to the full model at similar thresholds.

Our findings are consistent with previous studies highlighting the limitations of claims-based TED identification [4,5,13]. Stan et al. highlighted that the absence of a TED-specific ICD code limited accurate identification of TED in claims data, leading to potential misclassification, and incomplete capture of cases with prior Graves’ disease diagnoses [4]. Similarly, Patel et al. noted that the lack of a specific ICD-9 code for TED and reliance on claims data can lead to misclassification and underreporting of key clinical signs like lid retraction, which limited the accuracy and completeness of TED identification in claims-based analyses [5]. Our findings support the concerns, demonstrating that existing rule-based algorithms have limited sensitivity and may not reliably identify true TED cases. The lack of validated identification methods in earlier studies may have contributed to biased estimates of disease prevalence, treatment patterns, and outcomes. Our study helps address this gap by providing, to our knowledge, the first formal validation of TED identification algorithms in a linked real-world dataset.

Recent work has demonstrated that machine learning can enhance case identification for conditions lacking precise diagnostic codes [9,14,15,16]. Valdez et al. showed that machine learning can be used to improve myalgic encephalomyelitis/chronic fatigue syndrome case identification in large claims data [16]. Another study from Hara et al. showed that machine learning can effectively construct claims-based algorithms for chronic conditions such as hypertension, diabetes, and dyslipidemia, with a diagnostic accuracy comparable to traditional methods based on clinical experts’ expertise [9]. Similarly, Kural et al. demonstrated that both unsupervised and supervised machine learning methods could match or exceed expert rule-based algorithms in identifying anaphylaxis cases from claims data [15]. Our study extends this body of work by applying machine learning to a large, clinically enriched linked dataset and showing that machine learning-based models may offer additional flexibility for TED identification relative to traditional rule-based algorithms, although the magnitude of improvement was modest.

A key strength of this study is the use of a clinically validated reference standard derived from the IRIS Registry’s unstructured clinical notes, which enhances the reliability of our performance assessment. To our knowledge, this is the first study to validate TED identification algorithms against confirmed clinical documentation and to apply machine learning methods specifically for TED detection. The large, diverse patient population included in the IRIS Registry–Komodo linked dataset also strengthens the generalizability of our findings within the US healthcare system.

This study has several implications for observational studies in TED. Accurate patient identification is essential for valid estimation of disease burden, treatment patterns, and outcomes. The machine learning approach demonstrated improved sensitivity while preserving acceptable specificity and PPV, making it a viable tool for future observational studies using administrative data. Importantly, the ability to tune classification thresholds provides researchers with flexibility depending on whether higher sensitivity or specificity is required.

This study has several limitations. First, although the IRIS Registry NLP-derived classification provides a clinically informed reference standard, it is subject to misclassification, including a reported false positive rate of approximately 23%. In our study, false positive labeling of TED cases in the reference standard may lead to underestimation of sensitivity, as these cases are more likely to be classified as non-TED by the clinically informed algorithms and therefore counted as false negatives. This may partially contribute to the relatively low sensitivity observed in our results. Second, the high prevalence of TED in the analytic cohort (~72%) reflects the composition of the IRIS–Komodo linked validation dataset rather than population-level prevalence within the IRIS Registry, the full Komodo database, or a general Graves’ disease population. In this study, the Komodo data used for linkage did not represent the full Komodo population, but rather an enriched sample of patients with eye-related conditions. As a result, the analytic cohort had a substantially higher prevalence of TED than would be expected in a broader Graves’ disease or general claims population. Because positive and negative predictive values depend on disease prevalence, the PPV and NPV reported in this study may not directly generalize to lower-prevalence settings. In populations with lower TED prevalence, PPV would be expected to decrease and NPV to increase. Third, our study was restricted to the population captured in the IRIS Registry–Komodo linked dataset, which largely reflects patients seen in ophthalmology practices. This may limit generalizability to broader healthcare settings where patients with TED are also managed, such as primary care or endocrinology clinics. Unmeasured differences in disease severity, care-seeking behavior, referral patterns, and coding practices in those settings may differ, potentially affecting algorithm performance. Finally, an important limitation of this study is the lack of external validation in an independent dataset. We did not adjust for variation in coding practices across clinicians, regions, or healthcare systems, which may influence performance and consistency across different contexts. External validation in other populations will be necessary to further assess the generalizability and robustness of these findings.

In summary, rule-based algorithms demonstrated strong specificity and PPV and may be sufficient for many research applications. The CatBoost model, particularly in its simplified 29-code form, improved sensitivity while maintaining high specificity and PPV. Machine learning models provided additional flexibility through threshold tuning, allowing performance to be optimized based on specific study priorities. At selected thresholds, machine learning-based approaches improved sensitivity while maintaining high specificity and PPV. Using different thresholds, the models achieved very high specificity and PPV, though with a corresponding reduction in sensitivity. These trade-offs emphasize the importance of selecting thresholds that align with the specific objectives of the research. This adaptability makes machine learning a valuable complement to rule-based methods in claims-based observational studies.

## 5. Conclusions

This study validated the performance of existing rule-based algorithms for identifying TED cases in administrative claims data and highlighted the trade-offs between sensitivity and specificity. The machine learning model provided additional flexibility through threshold tuning, which allowed performance to align with different research priorities. The improvement over rule-based approaches was modest, suggesting that the potential value of machine learning may depend on the intended application. Although no single approach consistently outperformed others across all metrics, both rule-based and machine learning methods showed value in enhancing TED case identification using real-world data. Future research should include external validation and refinement of TED identification methods across diverse real-world datasets.

## Figures and Tables

**Table 1:** Description of algorithms for TED case identification.

Algorithm	Definition
Algorithm 1	Any claim with TED-related eye symptoms/signs including exophthalmos, diplopia, lid retraction, strabismus, orbital inflammation, ocular pain, keratoconjunctivitis, eye edema, visual disturbance, scotoma, vision deficiency, corneal ulcer, and optic neuropathy.
Algorithm 2	≥1 claim of hyperthyroidism along with ≥1 claim of TED-related eye symptoms/signs within 12 months. Symptoms include exophthalmos, diplopia, lid retraction, strabismus, and orbital inflammation.
Algorithm 3	Same as Algorithm 2 plus expanded symptoms: ocular pain and keratoconjunctivitis.
Algorithm 4	Same as Algorithm 2 plus broader symptoms: ocular pain, keratoconjunctivitis, eye edema, visual disturbance, scotoma, vision deficiency, corneal ulcer, and optic neuropathy.
Algorithm 5	Same as Algorithm 2 but different symptoms: exophthalmos, lid retraction, periorbital edema, and eyelid erythema.
Algorithm 6	Same as Algorithm 2, but without the 12-month timing restriction between hyperthyroidism and TED-related eye symptoms/signs.

TED: Thyroid Eye Disease. Codes for hyperthyroidism and TED-related eye symptoms/signs are available in Supplemental Table S1.

**Table 2:** Characteristics of TED Patients for Algorithm Validation.

	Linked Confirmed TED Patients (*N* = 66,396)		Linked Confirmed Non-TED Patients (*N* = 25,754) *	
	Komodo	IRIS	Komodo	IRIS
Characteristics	Mean (SD)/N(%)	Mean (SD)/N(%)	Mean (SD)/N(%)	Mean (SD)/N(%)
Age (Mean, SD)	60.4 (14.5)	60.4 (15.0)	58.8 (15.1)	58.8 (15.2)
Age group, n (%)				
18–30	2730 (4.1%)	2734 (4.1%)	1259 (4.9%)	1260 (4.9%)
31–40	5184 (7.8%)	5190 (7.8%)	2216 (8.6%)	2216 (8.6%)
41–50	8213 (12.4%)	8222 (12.4%)	3727 (14.5%)	3731 (14.5%)
51–60	13,723 (20.7%)	13,736 (20.7%)	5567 (21.6%)	5570 (21.6%)
61–70	17,889 (26.9%)	17,899 (27.0%)	6832 (26.5%)	6837 (26.5%)
71–80	13,962 (21.0%)	13,970 (21.0%)	4666 (18.1%)	4668 (18.1%)
80+	4695 (7.1%)	4645 (7.0%)	1487 (5.8%)	1472 (5.7%)
Gender, n (%)				
Male	15,260 (23.0%)	15,439 (23.3%)	5604 (21.8%)	5692 (22.1%)
Female	50,494 (76.0%)	50,957 (76.7%)	19,847 (77.1%)	20,062 (77.9%)
Unknown/missing	642 (1.0%)	0 (0.0%)	303 (1.2%)	0 (0.0%)
Race, n (%)				
White	N/A	37,807 (56.9%)	N/A	14,141 (54.9%)
Black or African American	N/A	5753 (8.7%)	N/A	2275 (8.8%)
Asian	N/A	1653 (2.5%)	N/A	565 (2.2%)
Other	N/A	21,183 (31.9%)	N/A	8773 (34.1%)
Insurance type				
Commercial	28,334 (42.7%)	8004 (12.1%)	12,262 (47.6%)	2361 (9.2%)
Medicare/Medicaid	37,895 (57.1%)	4259 (6.4%)	13,354 (51.9%)	1322 (5.1%)
Medicare/Medicaid combined with other	0 (0.0%)	3546 (5.3%)	0 (0.0%)	956 (3.7%)
Other/unknown	167 (0.3%)	50,587 (76.2%)	138 (0.5%)	21,115 (82.0%)
Charlson Comorbidity Index (mean ± sd)	2.7 (2.2)	5.1 (9.9)	2.0 (1.3)	4.7 (8.1)
Comorbidities				
Psoriasis	844 (1.3%)	57 (0.1%)	267 (1.0%)	32 (0.1%)
Psoriatic arthritis	325 (0.5%)	30 (0.0%)	119 (0.5%)	15 (0.1%)
Vitiligo	127 (0.2%)	0 (0.0%)	34 (0.1%)	0 (0.0%)
Systemic lupus erythematosus	603 (0.9%)	258 (0.4%)	214 (0.8%)	76 (0.3%)
Rheumatoid arthritis	1967 (3.0%)	451 (0.7%)	662 (2.6%)	142 (0.6%)
Myasthenia gravis	430 (0.6%)	534 (0.8%)	200 (0.8%)	187 (0.7%)
Crohn’s Disease	357 (0.5%)	45 (0.1%)	131 (0.5%)	7 (0.0%)
Ulcerative Colitis	419 (0.6%)	41 (0.1%)	174 (0.7%)	15 (0.1%)
Type 1 diabetes	1168 (1.8%)	688 (1.0%)	346 (1.3%)	136 (0.5%)
Type 2 diabetes	11,163 (16.8%)	7671 (11.6%)	3742 (14.5%)	2128 (8.3%)
Glaucoma	1133 (1.7%)	557 (0.8%)	387 (1.5%)	158 (0.6%)
Ocular hypertension	1127 (1.7%)	2793 (4.2%)	320 (1.2%)	592 (2.3%)
Dry eye	7391 (11.1%)	21,806 (32.8%)	2389 (9.3%)	5396 (21.0%)
Smoking or nicotine dependency	12,738 (19.2%)	832 (1.3%)	3750 (14.6%)	201 (0.8%)
Cannabis use disorders	551 (0.8%)	21 (0.0%)	168 (0.7%)	7 (0.0%)
Hypertension	26,853 (40.4%)	113 (0.2%)	9039 (35.1%)	36 (0.1%)
Obesity	17,916 (27.0%)	1869 (2.8%)	6164 (23.9%)	584 (2.3%)
COPD	6498 (9.8%)	372 (0.6%)	1943 (7.5%)	92 (0.4%)
CKD without dialysis	7610 (11.5%)	421 (0.6%)	2474 (9.6%)	134 (0.5%)
CKD with dialysis	261 (0.4%)	25 (0.0%)	108 (0.4%)	8 (0.0%)
Non-AD dementia	1000 (1.5%)	65 (0.1%)	347 (1.3%)	17 (0.1%)
Dyslipidemia	23,982 (36.1%)	1826 (2.8%)	8241 (32.0%)	560 (2.2%)

Abbreviations: TED, Thyroid Eye Disease; IRIS, Intelligent Research in Sight Registry; Komodo, Komodo Health Database; N, Number of patients; SD, Standard Deviation; COPD, Chronic Obstructive Pulmonary Disease; CKD, Chronic Kidney Disease; AD, Alzheimer’s Disease; N/A, Not Available. * For confirmed non-TED patients, a proxy index date was assigned based on the distribution of time intervals between the TED diagnosis date and the first record in IRIS data among TED patients.

**Table 3:** Codes used among the confirmed TED patients.

Code	Code Type	Code Description	Number of Patients (%)
Index Date ± 90 Days			
99214	CPT	Office/outpatient visit, established patient, moderate complexity	27,945 (42.1%)
99213	CPT	Office/outpatient visit, established patient, low complexity	24,812 (37.4%)
E05.00	ICD-10-CM	Thyrotoxicosis with diffuse goiter without thyrotoxic crisis	21,454 (32.3%)
36415	CPT	Collection of venous blood by venipuncture	16,539 (24.9%)
I10	ICD-10-CM	Essential (primary) hypertension	15,748 (23.7%)
84443	CPT	Thyroid stimulating hormone (TSH) test	12,631 (19.0%)
99204	CPT	Office/outpatient visit, new patient, moderate complexity	12,220 (18.4%)
80053	CPT	Comprehensive metabolic panel (CMP)	10,947 (16.5%)
85025	CPT	Complete blood count (CBC) with differential	10,798 (16.3%)
E03.9	ICD-10-CM	Hypothyroidism, unspecified	10,179 (15.3%)
84439	CPT	Free thyroxine (T4) test	9766 (14.7%)
E78.5	ICD-10-CM	Hyperlipidemia, unspecified	8159 (12.3%)
99203	CPT	Office/outpatient visit, new patient, low complexity	7877 (11.9%)
H04.123	ICD-10-CM	Dry eye syndrome of bilateral lacrimal glands	7824 (11.8%)
92014	CPT	Comprehensive eye examination, established patient	7817 (11.8%)
80061	CPT	Lipid panel	7495 (11.3%)
E05.90	ICD-10-CM	Thyrotoxicosis, unspecified without thyrotoxic crisis or storm	7357 (11.1%)
92083	CPT	Visual field examination, unilateral or bilateral	7118 (10.7%)
92012	CPT	Intermediate eye examination, established patient	7025 (10.6%)
H05.20	ICD-10-CM	Unspecified exophthalmos	6708 (10.1%)
Index date ± 180 days			
99214	CPT	Office/outpatient visit, established patient, moderate complexity	34,864 (52.5%)
99213	CPT	Office/outpatient visit, established patient, low complexity	32,713 (49.3%)
E05.00	ICD-10-CM	Thyrotoxicosis with diffuse goiter without thyrotoxic crisis	24,657 (37.1%)
36415	CPT	Collection of venous blood by venipuncture	21,696 (32.7%)
I10	ICD-10-CM	Essential (primary) hypertension	20,729 (31.2%)
99204	CPT	Office/outpatient visit, new patient, moderate complexity	16,873 (25.4%)
84443	CPT	Thyroid stimulating hormone (TSH) test	16,748 (25.2%)
80053	CPT	Comprehensive metabolic panel (CMP)	15,834 (23.9%)
85025	CPT	Complete blood count (CBC) with differential	15,727 (23.7%)
E03.9	ICD-10-CM	Hypothyroidism, unspecified	14,193 (21.4%)
84439	CPT	Free thyroxine (T4) test	12,891 (19.4%)
99203	CPT	Office/outpatient visit, new patient, low complexity	12,130 (18.3%)
E78.5	ICD-10-CM	Hyperlipidemia, unspecified	11,991 (18.1%)
80061	CPT	Lipid panel	11,744 (17.7%)
92014	CPT	Comprehensive eye examination, established patient	10,229 (15.4%)
Z00.00	ICD-10-CM	Encounter for general adult medical examination without abnormal findings	9873 (14.9%)
83036	CPT	Glycosylated hemoglobin (HbA1c) test	9564 (14.4%)
Z12.31	ICD-10-CM	Encounter for screening mammogram for malignant neoplasm of breast	9527 (14.4%)
E05.90	ICD-10-CM	Thyrotoxicosis, unspecified without thyrotoxic crisis or storm	9462 (14.3%)
H04.123	ICD-10-CM	Dry eye syndrome of bilateral lacrimal glands	9413 (14.2%)
Index date ± 365 days			
99214	CPT	Office/outpatient visit, established patient, moderate complexity	41,915 (63.1%)
99213	CPT	Office/outpatient visit, established patient, low complexity	40,805 (61.5%)
E05.00	ICD-10-CM	Thyrotoxicosis with diffuse goiter without thyrotoxic crisis	28,154 (42.4%)
36415	CPT	Collection of venous blood by venipuncture	27,613 (41.6%)
I10	ICD-10-CM	Essential (primary) hypertension	26,145 (39.4%)
99204	CPT	Office/outpatient visit, new patient, moderate complexity	23,620 (35.6%)
85025	CPT	Complete blood count (CBC) with differential	21,736 (32.7%)
80053	CPT	Comprehensive metabolic panel (CMP)	21,698 (32.7%)
84443	CPT	Thyroid stimulating hormone (TSH) test	21,651 (32.6%)
E03.9	ICD-10-CM	Hypothyroidism, unspecified	18,827 (28.4%)
99203	CPT	Office/outpatient visit, new patient, low complexity	18,744 (28.2%)
84439	CPT	Free thyroxine (T4) test	16,766 (25.3%)
80061	CPT	Lipid panel	16,625 (25.0%)
E78.5	ICD-10-CM	Hyperlipidemia, unspecified	16,554 (24.9%)
Z00.00	ICD-10-CM	Encounter for general adult medical examination without abnormal findings	14,949 (22.5%)
Z23	ICD-10-CM	Encounter for immunization	14,407 (21.7%)
Z12.31	ICD-10-CM	Encounter for screening mammogram for malignant neoplasm of breast	14,183 (21.4%)
92014	CPT	Comprehensive eye examination, established patient	13,918 (21.0%)
83036	CPT	Glycosylated hemoglobin (HbA1c) test	13,371 (20.1%)
Z79.899	ICD-10-CM	Long term (current) use of other medications	12,932 (19.5%)

Abbreviations: CPT, Current Procedural Terminology; ICD-10-CM, International Classification of Diseases, Tenth Revision, Clinical Modification.

**Table 4:** Performance metrics of rule-based TED algorithms.

	Sensitivity	Specificity	PPV	NPV	PLR	NLR	F1-Score
Algorithm 1	48.7% (48.3%, 49.1%)	59.9% (59.3%, 60.5%)	75.8% (75.4%, 76.2%)	31.2% (30.8%, 31.6%)	1.21 (1.19, 1.23)	0.86 (0.85, 0.87)	0.59 (0.59, 0.60)
Algorithm 2	23.6% (23.3%, 23.9%)	90.4% (90.1%, 90.8%)	86.4% (85.9%, 86.9%)	31.5% (31.1%, 31.8%)	2.47 (2.37, 2.57)	0.84 (0.84, 0.85)	0.37 (0.37, 0.37)
Algorithm 3	25.7% (25.4%, 26.0%)	88.5% (88.1%, 88.9%)	85.2% (84.7%, 85.7%)	31.6% (31.3%, 31.9%)	2.24 (2.16, 2.32)	0.84 (0.83, 0.84)	0.39 (0.39, 0.40)
Algorithm 4	27.0% (26.7%, 27.4%)	87.2% (86.8%, 87.6%)	84.5% (84.0%, 84.9%)	31.7% (31.3%, 32.0%)	2.11 (2.04, 2.18)	0.84 (0.83, 0.84)	0.41 (0.41, 0.41)
Algorithm 5	17.8% (17.5%, 18.1%)	93.5% (93.2%, 93.8%)	87.6% (87.1%, 88.2%)	30.6% (30.3%, 30.9%)	2.74 (2.61, 2.88)	0.88 (0.87, 0.88)	0.30 (0.29, 0.30)
Algorithm 6	33.4% (33.1%, 33.8%)	86.8% (86.4%, 87.2%)	86.7% (86.3%, 87.1%)	33.6% (33.2%, 33.9%)	2.54 (2.45, 2.62)	0.77 (0.76, 0.77)	0.48 (0.48, 0.49)

Abbreviations: TED, Thyroid Eye Disease; PPV, Positive Predictive Value; NPV, Negative Predictive Value; PLR, Positive Likelihood Ratio; NLR, Negative Likelihood Ratio; F1-score, harmonic mean of precision and recall. Values in parentheses represent 95% confidence intervals.

**Table 5:** Diagnosis and procedure codes included in the final simplified feature set for machine learning-based TED identification.

Codes	Code Type	Description
92004	CPT	Comprehensive ophthalmological service for a new patient
E05.00	ICD-10-CM	Thyrotoxicosis with diffuse goiter without thyrotoxic crisis or storm
92285	CPT	External ocular photography with interpretation and report
H05.20	ICD-10-CM	Unspecified exophthalmos
E05.90	ICD-10-CM	Thyrotoxicosis, unspecified without thyrotoxic crisis or storm
H05.243	ICD-10-CM	Constant exophthalmos, bilateral
92136	CPT	Under Ophthalmological Examination and Evaluation Procedures
H53.2	ICD-10-CM	Diplopia
92250	CPT	Under Ophthalmoscopy Procedures
92083	CPT	Under Ophthalmological Examination and Evaluation Procedures
92133	CPT	Under Ophthalmological Examination and Evaluation Procedures
66984	CPT	Under Intraocular Lens Procedures
H25.13	ICD-10-CM	Age-related nuclear cataract, bilateral
E07.9	ICD-10-CM	Disorder of thyroid, unspecified.
76514	CPT	Ophthalmic ultrasound, diagnostic; corneal pachymetry, unilateral or bilateral (determination of corneal thickness)
92002	CPT	Ophthalmological services: medical examination and evaluation with initiation of diagnostic and treatment program; new patient
E03.9	ICD-10-CM	Hypothyroidism, Unspecified
92015	CPT	Determination of refractive state
H57.89	ICD-10-CM	Other Specified Disorders of Eye and Adnexa
H02.531	ICD-10-CM	Eyelid Retraction, Right Upper Eyelid
92060	CPT	Sensorimotor examination with multiple measurements of ocular deviation (e.g., restrictive or paretic muscle with diplopia); with interpretation and report
92014	CPT	Ophthalmological services; comprehensive, established patient
78014	CPT	Thyroid scan and uptake; including suppression
84481	CPT	Calcitonin (thyroid hormone) level
H05.89	ICD-10-CM	Other disorders of orbit
E05.01	ICD-10-CM	Thyrotoxicosis with diffuse goiter with thyrotoxic crisis or storm
H16.223	ICD-10-CM	Keratoconjunctivitis sicca, not specified as Sjögren’s, bilateral
92012	CPT	Ophthalmological services: medical examination and evaluation, established patient, with initiation or continuation of diagnostic and treatment program
E89.0	ICD-10-CM	Postprocedural hypothyroidism

Abbreviations: CPT, Current Procedural Terminology; ICD-10, International Classification of Diseases, Tenth Revision, Clinical Modification.

**Table 6:** Performance of the full and simplified CatBoost models for TED identification across probability thresholds.

Probability Threshold	Sensitivity	Specificity	PPV	NPV	PLR	NLR	F1-Score
Full CatBoost model							
0.60	86.8% (86.4%, 87.1%)	29.4% (28.6%, 30.3%)	76.0% (75.6%, 76.5%)	46.3% (45.2%, 47.3%)	1.23 (1.22, 1.25)	0.45 (0.43, 0.47)	0.81 (0.81, 0.81)
0.65	70.8% (70.3%, 71.3%)	55.4% (54.5%, 56.2%)	80.4% (79.9%, 80.8%)	42.4% (41.7%, 43.1%)	1.59 (1.56, 1.62)	0.53 (0.52, 0.54)	0.75 (0.75, 0.76)
0.70	57.9% (57.4%, 58.4%)	70.3% (69.4%, 71.1%)	83.4% (82.9%, 83.9%)	39.3% (38.6%, 39.9%)	1.95 (1.89, 2.01)	0.60 (0.59, 0.61)	0.68 (0.68, 0.69)
0.75	49.0% (48.4%, 49.5%)	78.4% (77.6%, 79.1%)	85.4% (84.9%, 85.9%)	37.3% (36.7%, 37.9%)	2.27 (2.19, 2.34)	0.65 (0.64, 0.66)	0.62 (0.62, 0.63)
0.80	40.1% (39.6%, 40.7%)	85.4% (84.8%, 86.0%)	87.6% (87.1%, 88.1%)	35.6% (35.1%, 36.1%)	2.75 (2.63, 2.87)	0.70 (0.69, 0.71)	0.55 (0.55, 0.56)
0.85	27.7% (27.3%, 28.2%)	92.5% (92.0%, 92.9%)	90.5% (89.9%, 91.1%)	33.2% (32.7%, 33.6%)	3.69 (3.48, 3.94)	0.78 (0.78, 0.79)	0.43 (0.42, 0.43)
Simplified CatBoost model							
0.60	70.1% (69.6%, 70.6%)	51.2% (50.3%, 52.1%)	78.8% (78.3%, 79.2%)	39.9% (39.2%, 40.6%)	1.44 (1.41, 1.47)	0.58 (0.57, 0.60)	0.74 (0.74, 0.75)
0.65	61.9% (61.4%, 62.4%)	63.0% (62.1%, 63.8%)	81.2% (80.6%, 81.7%)	39.0% (38.3%, 39.7%)	1.67 (1.63, 1.71)	0.61 (0.59, 0.62)	0.70 (0.70, 0.71)
0.70	56.7% (56.1%, 57.2%)	69.4% (68.6%, 70.2%)	82.7% (82.2%, 83.2%)	38.3% (37.7%, 38.9%)	1.85 (1.80, 1.91)	0.62 (0.61, 0.64)	0.67 (0.67, 0.68)
0.75	49.3% (48.7%, 49.8%)	77.2% (76.4%, 77.9%)	84.8% (84.3%, 85.3%)	37.1% (36.5%, 37.7%)	2.16 (2.09, 2.23)	0.66 (0.65, 0.67)	0.62 (0.62, 0.63)
0.80	39.3% (38.8%, 39.8%)	84.8% (84.1%, 85.4%)	86.9% (86.4%, 87.5%)	35.1% (34.6%, 35.6%)	2.58 (2.47, 2.70)	0.72 (0.71, 0.72)	0.54 (0.54, 0.55)
0.85	28.5% (28.0%, 29.0%)	91.7% (91.2%, 92.1%)	89.8% (89.3%, 90.4%)	33.2% (32.7%, 33.7%)	3.43 (3.24, 3.64)	0.78 (0.77, 0.79)	0.43 (0.43, 0.44)

Abbreviations: TED, Thyroid Eye Disease; PPV, Positive Predictive Value; NPV, Negative Predictive Value; PLR, Positive Likelihood Ratio; NLR, Negative Likelihood Ratio; F1-score, harmonic mean of precision and recall. Cutoff refers to the predicted probability threshold used to classify patients as TED or non-TED. Values in parentheses indicate 95% confidence intervals.

## Data Availability

Restrictions apply to the availability of these data. Data were obtained from the American Academy of Ophthalmology (AAO) IRIS^®^ Registry and the Komodo Health^®^ Research Dataset and are not publicly available due to data use agreements and privacy restrictions. Access to the data may be requested directly from the respective data providers (AAO IRIS^®^ Registry and Komodo Health^®^), subject to their approval and licensing requirements.

## Supplementary Materials

The following supporting information can be downloaded at: https://www.mdpi.com/article/10.3390/jcm15103836/s1, Figure S1: Patient flow diagram for study cohort; Table S1: ICD-10 codes for hyperthyroidism and TED–related eye symptoms and signs.

## Abbreviations

The following abbreviations are used in this manuscript:

AAO	American Academy of Ophthalmology
AD	Alzheimer’s Disease
AUC	Area Under the Curve
CatBoost	Categorical Boosting (a gradient boosting algorithm)
CCI	Charlson Comorbidity Index
CKD	Chronic Kidney Disease
COPD	Chronic Obstructive Pulmonary Disease
CPT	Current Procedural Terminology
GD	Graves’ Disease
EHR	Electronic Health Record
HCPCS	Healthcare Common Procedure Coding System
ICD	International Classification of Diseases
IRIS	Intelligent Research in Sight (IRIS^®^ Registry)
NDC	National Drug Code
NLP	Natural Language Processing
NPV	Negative Predictive Value
PPV	Positive Predictive Value
RFE	Recursive Feature Elimination
TED	Thyroid Eye Disease
US	United States
