# Supplementary material for: Validation and Development of Claims-Based Algorithms for Identifying Thyroid Eye Disease Using the IRIS Registry-Komodo Linked Database
Source: J Clin Med. 2026 May 15;15(10):3836. doi: 10.3390/jcm15103836 (PMC13207259; doi:10.3390/jcm15103836)
Supplement: Supplementary file 1 [file jcm-15-03836-s001.zip › Supplemental Table S1.pdf]

Supplemental Table S1. ICD-10 codes for hyperthyroidism and TED–related eye symptoms and signs

Criterion Name	Code Type	Code	Code Description
Hyperthyroidism	ICD-10-CM Diagnosis	E05	thyrotoxicosis
	ICD-10-CM Diagnosis	E050	thyrotoxicosis with diffuse goiter
	ICD-10-CM Diagnosis	E0500	thyrotoxicosis with diffuse goiter without thyrotoxic crisis or storm
	ICD-10-CM Diagnosis	E0501	thyrotoxicosis with diffuse goiter with thyrotoxic crisis or storm
	ICD-10-CM Diagnosis	E0510	thyrotoxicosis with toxic single thyroid nodule without thyrotoxic crisis or storm
	ICD-10-CM Diagnosis	E0511	thyrotoxicosis with toxic single thyroid nodule with thyrotoxic crisis or storm
	ICD-10-CM Diagnosis	E0520	thyrotoxicosis with toxic multinodular goiter without thyrotoxic crisis or storm
	ICD-10-CM Diagnosis	E0521	thyrotoxicosis with toxic multinodular goiter with thyrotoxic crisis or storm
	ICD-10-CM Diagnosis	E0530	thyrotoxicosis from ectopic thyroid tissue without thyrotoxic crisis or storm
	ICD-10-CM Diagnosis	E0531	thyrotoxicosis from ectopic thyroid tissue with thyrotoxic crisis or storm
	ICD-10-CM Diagnosis	E0540	thyrotoxicosis factitia without thyrotoxic crisis or storm
	ICD-10-CM Diagnosis	E0541	thyrotoxicosis factitia with thyrotoxic crisis or storm

	ICD-10-CM Diagnosis	E0580	other thyrotoxicosis without thyrotoxic crisis or storm
	ICD-10-CM Diagnosis	E0581	other thyrotoxicosis with thyrotoxic crisis or storm
	ICD-10-CM Diagnosis	E059	thyrotoxicosis, unspecified
	ICD-10-CM Diagnosis	E0590	thyrotoxicosis unspecified without thyrotoxic crisis or storm
	ICD-10-CM Diagnosis	E0591	thyrotoxicosis unspecified with thyrotoxic crisis or storm
	ICD-10-CM Diagnosis	E062	Chronic thyroiditis with transient thyrotoxicosis
Exophthalmos	ICD-10-CM Diagnosis	H05213	displacement (lateral) of globe bilateral
	ICD-10-CM Diagnosis	H05212	displacement (lateral) of globe left eye
	ICD-10-CM Diagnosis	H05211	displacement (lateral) of globe right eye
	ICD-10-CM Diagnosis	H05219	displacement (lateral) of globe unspecified eye
	ICD-10-CM Diagnosis	H05342	enlargement of left orbit
	ICD-10-CM Diagnosis	H05341	enlargement of right orbit
	ICD-10-CM Diagnosis	H05349	enlargement of unspecified orbit
	ICD-10-CM Diagnosis	H05259	intermittent exophthalmos unspecified eye
	ICD-10-CM Diagnosis	H05269	pulsating exophthalmos unspecified eye
	ICD-10-CM Diagnosis	H05240	constant exophthalmos
	ICD-10-CM Diagnosis	H05243	constant exophthalmos bilateral
	ICD-10-CM Diagnosis	H05242	constant exophthalmos left eye
	ICD-10-CM Diagnosis	H05241	constant exophthalmos right eye
	ICD-10-CM Diagnosis	H05249	constant exophthalmos unspecified eye

	ICD-10-CM Diagnosis	H0520	unspecified exophthalmos
Diplopia	ICD-10-CM Diagnosis	H532	Diplopia
Lid retraction	ICD-10-CM Diagnosis	H0220C	bilateral, upper and lower eyelids lagophthalmos
	ICD-10-CM Diagnosis	H0221	cicatricial lagophthalmos
	ICD-10-CM Diagnosis	H0221C	cicatricial lagophthalmos, bilateral, upper and lower eyelids
	ICD-10-CM Diagnosis	H02216	cicatricial lagophthalmos, left eye, unspecified eyelid
	ICD-10-CM Diagnosis	H0221B	cicatricial lagophthalmos, left eye, upper and lower eyelids
	ICD-10-CM Diagnosis	H02215	cicatricial lagophthalmos, left lower eyelid
	ICD-10-CM Diagnosis	H02214	cicatricial lagophthalmos, left upper eyelid
	ICD-10-CM Diagnosis	H02213	cicatricial lagophthalmos, right eye, unspecified eyelid
	ICD-10-CM Diagnosis	H0221A	cicatricial lagophthalmos, right eye, upper and lower eyelids
	ICD-10-CM Diagnosis	H02212	cicatricial lagophthalmos, right lower eyelid
	ICD-10-CM Diagnosis	H02211	cicatricial lagophthalmos, right upper eyelid
	ICD-10-CM Diagnosis	H02219	cicatricial lagophthalmos, unspecified eye, unspecified eyelid
	ICD-10-CM Diagnosis	H0253	eyelid retraction
	ICD-10-CM Diagnosis	H02536	eyelid retraction left eye, unspecified eyelid
	ICD-10-CM Diagnosis	H02535	eyelid retraction left lower eyelid
	ICD-10-CM Diagnosis	H02534	eyelid retraction left upper eyelid
	ICD-10-CM Diagnosis	H02533	eyelid retraction right eye, unspecified eyelid

	ICD-10-CM Diagnosis	H02532	eyelid retraction right lower eyelid
	ICD-10-CM Diagnosis	H02531	eyelid retraction right upper eyelid
	ICD-10-CM Diagnosis	H02539	eyelid retraction unspecified eye unspecified lid
	ICD-10-CM Diagnosis	H022	lagophthalmos
	ICD-10-CM Diagnosis	H0220B	left eye, upper and lower eyelids lagophthalmos
	ICD-10-CM Diagnosis	H0222	mechanical lagophthalmos
	ICD-10-CM Diagnosis	H02229	mechanical lagophthalmos unspecified eye, unspecified eyelid
	ICD-10-CM Diagnosis	H0222C	mechanical lagophthalmos, bilateral, upper and lower eyelids
	ICD-10-CM Diagnosis	H02226	mechanical lagophthalmos, left eye, unspecified eyelid
	ICD-10-CM Diagnosis	H0222B	mechanical lagophthalmos, left eye, upper and lower eyelids
	ICD-10-CM Diagnosis	H02225	mechanical lagophthalmos, left lower eyelid
	ICD-10-CM Diagnosis	H02224	mechanical lagophthalmos, left upper eyelid
	ICD-10-CM Diagnosis	H02223	mechanical lagophthalmos, right eye, unspecified eyelid
	ICD-10-CM Diagnosis	H0222A	mechanical lagophthalmos, right eye, upper and lower eyelids
	ICD-10-CM Diagnosis	H02222	mechanical lagophthalmos, right lower eyelid
	ICD-10-CM Diagnosis	H02221	mechanical lagophthalmos, right upper eyelid
	ICD-10-CM Diagnosis	H0223	Paralytic lagophthalmos

	ICD-10-CM Diagnosis	H0223C	Paralytic lagophthalmos, bilateral, upper and lower eyelids
	ICD-10-CM Diagnosis	H02236	Paralytic lagophthalmos, left eye, unspecified eyelid
	ICD-10-CM Diagnosis	H0223B	Paralytic lagophthalmos, left eye, upper and lower eyelids
	ICD-10-CM Diagnosis	H02235	Paralytic lagophthalmos, left lower eyelid
	ICD-10-CM Diagnosis	H02234	Paralytic lagophthalmos, left upper eyelid
	ICD-10-CM Diagnosis	H02233	Paralytic lagophthalmos, right eye, unspecified eyelid
	ICD-10-CM Diagnosis	H0223A	Paralytic lagophthalmos, right eye, upper and lower eyelids
	ICD-10-CM Diagnosis	H02232	Paralytic lagophthalmos, right lower eyelid
	ICD-10-CM Diagnosis	H02231	Paralytic lagophthalmos, right upper eyelid
	ICD-10-CM Diagnosis	H02239	Paralytic lagophthalmos, unspecified eye, unspecified eyelid
	ICD-10-CM Diagnosis	H0220A	right eye, upper and lower eyelids lagophthalmos
	ICD-10-CM Diagnosis	H0220	unspecified lagophthalmos
	ICD-10-CM Diagnosis	H02206	unspecified lagophthalmos left eye unspecified eyelid
	ICD-10-CM Diagnosis	H02205	unspecified lagophthalmos left lower eyelid
	ICD-10-CM Diagnosis	H02204	unspecified lagophthalmos left upper eyelid
	ICD-10-CM Diagnosis	H02203	unspecified lagophthalmos right eye unspecified eyelid
	ICD-10-CM Diagnosis	H02202	unspecified lagophthalmos right lower eyelid

	ICD-10-CM Diagnosis	H02201	unspecified lagophthalmos right upper eyelid
	ICD-10-CM Diagnosis	H02209	unspecified lagophthalmos unspecified eye, unspecified eyelid
Strabismus	ICD-10-CM Diagnosis	H5043	accommodative component in esotropia
	ICD-10-CM Diagnosis	H5005	alternating esotropia
	ICD-10-CM Diagnosis	H5006	alternating esotropia with a pattern
	ICD-10-CM Diagnosis	H5008	alternating esotropia with other noncomitancies
	ICD-10-CM Diagnosis	H5007	alternating esotropia with v pattern
	ICD-10-CM Diagnosis	H5015	alternating exotropia
	ICD-10-CM Diagnosis	H5016	alternating exotropia with a pattern
	ICD-10-CM Diagnosis	H5018	alternating exotropia with other noncomitancies
	ICD-10-CM Diagnosis	H5017	alternating exotropia with v pattern
	ICD-10-CM Diagnosis	H5041	cyclotropia
	ICD-10-CM Diagnosis	H50412	cyclotropia, left eye
	ICD-10-CM Diagnosis	H50411	cyclotropia, right eye
	ICD-10-CM Diagnosis	H500	Esotropia
	ICD-10-CM Diagnosis	H501	exotropia
	ICD-10-CM Diagnosis	H505	heterotropia
	ICD-10-CM Diagnosis	H5032	intermittent alternating esotropia
	ICD-10-CM Diagnosis	H5034	intermittent alternating exotropia
	ICD-10-CM Diagnosis	H503	intermittent heterotropia
	ICD-10-CM Diagnosis	H50312	intermittent monocular esotropia, left eye

	ICD-10-CM Diagnosis	H50311	intermittent monocular esotropia, right eye
	ICD-10-CM Diagnosis	H5033	intermittent monocular exotropia
	ICD-10-CM Diagnosis	H50332	intermittent monocular exotropia, left eye
	ICD-10-CM Diagnosis	H50331	intermittent monocular exotropia, right eye
	ICD-10-CM Diagnosis	H506	Mechanical strabismus
	ICD-10-CM Diagnosis	H5060	mechanical strabismus unspecified
	ICD-10-CM Diagnosis	H5001	Monocular esotropia
	ICD-10-CM Diagnosis	H5002	monocular esotropia with a pattern
	ICD-10-CM Diagnosis	H50022	monocular esotropia with a pattern, left eye
	ICD-10-CM Diagnosis	H50021	monocular esotropia with a pattern, right eye
	ICD-10-CM Diagnosis	H5004	monocular esotropia with other noncomitancies
	ICD-10-CM Diagnosis	H50042	monocular esotropia with other noncomitancies, left eye
	ICD-10-CM Diagnosis	H50041	monocular esotropia with other noncomitancies, right eye
	ICD-10-CM Diagnosis	H5003	monocular esotropia with v pattern
	ICD-10-CM Diagnosis	H50032	monocular esotropia with v pattern, left eye
	ICD-10-CM Diagnosis	H50031	monocular esotropia with v pattern, right eye
	ICD-10-CM Diagnosis	H50012	monocular esotropia, left eye
	ICD-10-CM Diagnosis	H50011	monocular esotropia, right eye
	ICD-10-CM Diagnosis	H5011	monocular exotropia

	ICD-10-CM Diagnosis	H5012	monocular exotropia with a pattern
	ICD-10-CM Diagnosis	H50122	monocular exotropia with a pattern, left eye
	ICD-10-CM Diagnosis	H50121	monocular exotropia with a pattern, right eye
	ICD-10-CM Diagnosis	H5014	monocular exotropia with other noncomitancies
	ICD-10-CM Diagnosis	H50142	monocular exotropia with other noncomitancies, left eye
	ICD-10-CM Diagnosis	H50141	monocular exotropia with other noncomitancies, right eye
	ICD-10-CM Diagnosis	H5013	monocular exotropia with v pattern
	ICD-10-CM Diagnosis	H50132	monocular exotropia with v pattern, left eye
	ICD-10-CM Diagnosis	H50131	monocular exotropia with v pattern, right eye
	ICD-10-CM Diagnosis	H50112	monocular exotropia, left eye
	ICD-10-CM Diagnosis	H50111	monocular exotropia, right eye
	ICD-10-CM Diagnosis	H5042	monofixation syndrome
	ICD-10-CM Diagnosis	H504	other and unspecified heterotropia
	ICD-10-CM Diagnosis	H5069	other mechanical strabismus
	ICD-10-CM Diagnosis	H4988	other paralytic strabismus
	ICD-10-CM Diagnosis	H49889	other paralytic strabismus, bilateral
	ICD-10-CM Diagnosis	H49883	other paralytic strabismus, left eye
	ICD-10-CM Diagnosis	H49882	other paralytic strabismus, right eye
	ICD-10-CM Diagnosis	H49881	other paralytic strabismus, unspecified eye

	ICD-10-CM Diagnosis	H5089	Other specified strabismus
	ICD-10-CM Diagnosis	H49	paralytic strabismus
	ICD-10-CM Diagnosis	H5000	unspecified esotropia
	ICD-10-CM Diagnosis	H5010	unspecified exotropia
	ICD-10-CM Diagnosis	H5050	Unspecified heterophoria
	ICD-10-CM Diagnosis	H5040	unspecified heterotropia
	ICD-10-CM Diagnosis	H5030	unspecified intermittent heterotropia
	ICD-10-CM Diagnosis	H499	unspecified paralytic strabismus
	ICD-10-CM Diagnosis	H509	unspecified strabismus
	ICD-10-CM Diagnosis	H502	vertical strabismus
	ICD-10-CM Diagnosis	H5022	vertical strabismus left eye
	ICD-10-CM Diagnosis	H5021	vertical strabismus right eye
Orbital inflammation/disorders	ICD-10-CM Diagnosis	H47093	other disorders of optic nerve, bilateral
	ICD-10-CM Diagnosis	H47092	other disorders of optic nerve, left eye
	ICD-10-CM Diagnosis	H4709	other disorders of optic nerve, not elsewhere classified
	ICD-10-CM Diagnosis	H47099	other disorders of optic nerve, not elsewhere classified, unspecified eye
	ICD-10-CM Diagnosis	H47091	other disorders of optic nerve, right eye
	ICD-10-CM Diagnosis	H0589	other disorders of orbit
	ICD-10-CM Diagnosis	H578	other specified disorders of eye and adnexa
	ICD-10-CM Diagnosis	H5334	suppression of binocular vision
	ICD-10-CM Diagnosis	H0500	unspecified acute inflammation of orbit
	ICD-10-CM Diagnosis	H0510	unspecified chronic inflammatory disorders of orbit

	ICD-10-CM Diagnosis	H5330	unspecified disorder of binocular vision
	ICD-10-CM Diagnosis	H579	unspecified disorder of eye and adnexa
