# Supplementary figures and images for: Validation and Development of Claims-Based Algorithms for Identifying Thyroid Eye Disease Using the IRIS Registry-Komodo Linked Database
Source: J Clin Med. 2026 May 15;15(10):3836. doi: 10.3390/jcm15103836 (PMC13207259; doi:10.3390/jcm15103836)

**Supplemental Figure S1. Patient flow diagram for study cohort**

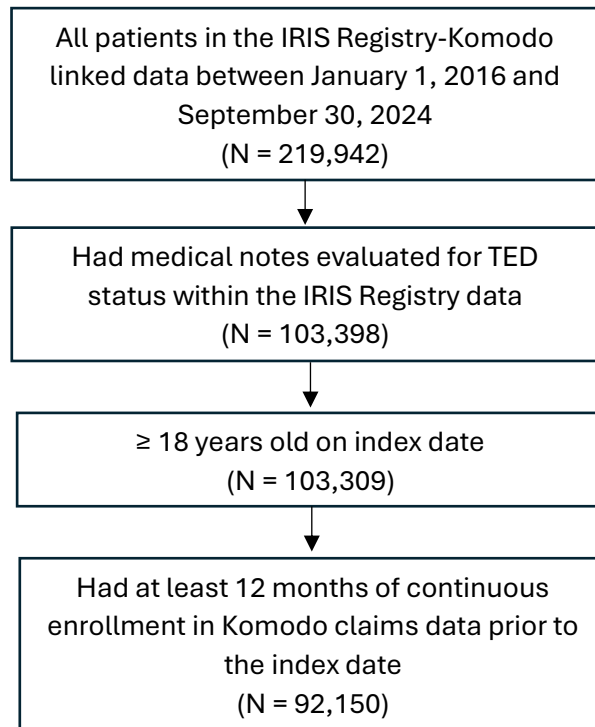

Supplement: Supplementary file 1 [file jcm-15-03836-s001.zip › Supplemental Figure S1_10092025.pdf]
